# Effect of Chronic Treatment with Uridine on Cardiac Mitochondrial Dysfunction in the C57BL/6 Mouse Model of High-Fat Diet–Streptozotocin-Induced Diabetes

**DOI:** 10.3390/ijms231810633

**Published:** 2022-09-13

**Authors:** Natalia V. Belosludtseva, Vlada S. Starinets, Irina B. Mikheeva, Maxim N. Belosludtsev, Mikhail V. Dubinin, Galina D. Mironova, Konstantin N. Belosludtsev

**Affiliations:** 1 Laboratory of Mitochondrial Transport, Institute of Theoretical and Experimental Biophysics, Russian Academy of Sciences, Institutskaya 3, Pushchino 142290, Russia; 2 Department of Biochemistry, Cell Biology and Microbiology, Mari State University, pl. Lenina 1, Yoshkar-Ola 424001, Russia

**Keywords:** mitochondria, diabetes mellitus, uridine, mitochondrial dysfunction, lipid peroxidation, mitochondrial biogenesis

## Abstract

Long-term hyperglycemia in diabetes mellitus is associated with complex damage to cardiomyocytes and the development of mitochondrial dysfunction in the myocardium. Uridine, a pyrimidine nucleoside, plays an important role in cellular metabolism and is used to improve cardiac function. Herein, the antidiabetic potential of uridine (30 mg/kg/day for 21 days, i.p.) and its effect on mitochondrial homeostasis in the heart tissue were examined in a high-fat diet–streptozotocin-induced model of diabetes in C57BL/6 mice. We found that chronic administration of uridine to diabetic mice normalized plasma glucose and triglyceride levels and the heart weight/body weight ratio and increased the rate of glucose utilization during the intraperitoneal glucose tolerance test. Analysis of TEM revealed that uridine prevented diabetes-induced ultrastructural abnormalities in mitochondria and sarcomeres in ventricular cardiomyocytes. In diabetic heart tissue, the mRNA level of *Ppargc1a* decreased and *Drp1* and *Parkin* gene expression increased, suggesting the disturbances of mitochondrial biogenesis, fission, and mitophagy, respectively. Uridine treatment of diabetic mice restored the mRNA level of *Ppargc1a* and enhanced *Pink1* gene expression, which may indicate an increase in the intensity of mitochondrial biogenesis and mitophagy, and as a consequence, mitochondrial turnover. Uridine also reduced oxidative phosphorylation dysfunction and suppressed lipid peroxidation, but it had no significant effect on the impaired calcium retention capacity and potassium transport in the heart mitochondria of diabetic mice. Altogether, these findings suggest that, along with its hypoglycemic effect, uridine has a protective action against diabetes-mediated functional and structural damage to cardiac mitochondria and disruption of mitochondrial quality-control systems in the diabetic heart.

## 1. Introduction

Diabetes mellitus (DM) is a group of metabolic disorders of different etiologies that are associated with insufficient secretion of insulin from the β-cells of the pancreatic islets and variable degrees of peripheral insulin resistance, resulting in hyperglycemia [1]. The search for new approaches to the treatment of DM is an urgent task, since the disease has become a global pandemic and a serious health-care burden worldwide. According to the International Diabetes Federation, in 2021 there were 537 million people with diabetes, i.e., 10% of the world’s population, and about 50% of all patients with diabetes were in the most active working age of 40–59 years [2]. Complications of diabetes include damage to target organs, mainly the heart, as one of the body’s most energy-demanding organs [3]. Diabetic cardiomyopathy manifests itself in significant abnormalities of both the structure and functions of cardiomyocytes, which may ultimately lead to the development of myocardial ischemic injury, heart attack, and death of diabetic patients [4,5]. The cellular and molecular mechanisms contributing to the development of diabetic cardiomyopathy involve, but are not limited to, cardiomyocyte glucotoxicity and lipotoxicity, damage from glycated end products and ROS, altered substrate utilization and energy metabolism, impaired calcium homeostasis, and mitochondrial dysfunction in the heart tissue [4,5,6,7,8]. The complexity of diabetic heart injury suggests that the targeting of multiple mechanisms may have a broader therapeutic efficacy.

Uridine is a pyrimidine nucleoside that plays an important role in maintaining cellular function and energy metabolism [9]. Uridine is a building block for RNA biosynthesis and is involved in the processes of cell signaling. It also required for protein and lipid glycosylation, extracellular matrix biosynthesis, and the metabolic clearance of xenobiotics. As the UTP precursor, uridine can activate glycogen synthesis [9,10,11]. Uridine metabolism is closely associated with glucose homeostasis, lipid metabolism, and amino acid exchange by regulating key enzymes and their reaction products, such as UTP, dihydroorotate dehydrogenase, and uridine phosphorylase, which are then involved in systemic metabolism [9,10,11,12].

Uridine and its derivatives have been widely used to reduce cytotoxicity, suppress drug-induced hepatic steatosis, and improve neurophysiological functions [9,13,14,15]. Our previous studies showed that uridine administration prevents myocardial injury in rat models of acute ischemia and ischemia–reperfusion by restoring redox balance and activating the mitochondrial ATP-dependent potassium channel [16,17,18,19]. Recent data suggest that uridine can regulate the functioning of the mitochondrial respiratory chain [12]. Therefore, the control of uridine levels in plasma and tissues can be coupled with mitochondrial function and systemic energy homeostasis.

At the same time, data on the effects of uridine on the progression of diabetes mellitus and insulin-resistant states are rather contradictory. On the one hand, oral administration of uridine with food was shown to improve insulin sensitivity in healthy mice and animals kept on a high-fat diet [20,21,22]. Long-term use of uridine in diabetic patients resulted in an improvement in neurophysiological parameters [23]. On the other hand, some studies revealed that long-term supplementation with uridine had opposing effects on liver lipid metabolism and could promote the development of prediabetes in mice [12,24]. The effect of uridine treatment on diabetes-induced changes in the heart has not yet been studied sufficiently. It was demonstrated that plasma uridine concentration in patients with type 1 and type 2 diabetes was higher than that in healthy individuals [25,26]. Contrariwise, mice with experimental type 1 diabetes showed decreased levels of uridine in plasma, bile, brown adipose tissue, and heart tissue [20].

Given the beneficial effects of uridine on cell-energy metabolism and redox status, we suggested that the nucleoside could serve as a supplemental therapy to ameliorate metabolic disorders associated with mitochondrial alterations. Mitochondrial dysfunction is known to play an important role in both the development and progression of DM, particularly due to excessive production of ROS, a loss of mitochondrial membrane potential, impaired oxidative phosphorylation, and increased susceptibility of the organelles to the permeability transition pore (PTP) opening, which eventually could lead to mitochondrial damage. In parallel, DM is accompanied by a malfunction of the mitochondrial quality-control system and a disruption of mitochondrial dynamics and mitochondrial biogenesis, resulting in the accumulation of defects in the mitochondrial network and the dysregulation of cellular energy supply [8,27,28]. It is important to note that some pharmacological agents capable of normalizing mitochondrial function can attenuate the course of diabetes [29,30].

Taking into account the above, the aim of the present work was to evaluate the antidiabetic potential of uridine (30 mg/kg/day for 21 days, i.p.) and to study the effect of the nucleoside on the development of mitochondrial dysfunction in the heart tissue in the C57BL/6 mouse model of a high-fat diet–streptozotocin-induced DM.

## 2. Results

### 2.1. Effect of Uridine on Somatic and Biochemical Indices of C57BL/6 Mice

**Figure 1:**
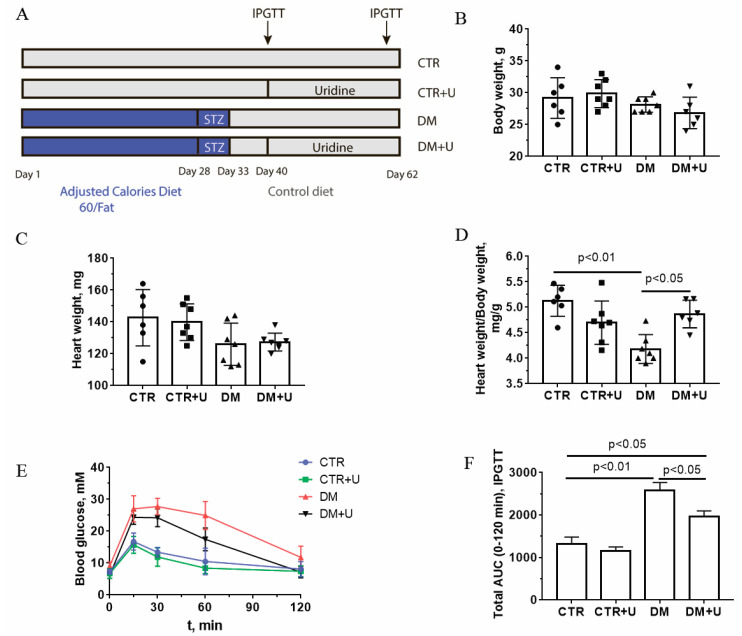
Scheme for induction of diabetes mellitus (**A**), body weight (**B**), absolute (**C**) and relative heart weight (**D**), intraperitoneal glucose tolerance test, IPGTT (**E**) in control (CTR), uridine-treated control (CTR + U), diabetic (DM), and uridine-treated diabetic (DM + U) C57BL/6 mice. The total areas under the curve (AUC) of the IPGTT (**F**). The tests were conducted on the 60th day from the beginning of the experiment. All data are presented as means ± SD (*n* = 5–7).

The design of the experiments and the scheme for induction of DM are outlined in Figure 1A. Uridine at a dose of 30 mg/kg was administered intraperitoneally to control and diabetic mice for 21 days. The dose and regimen were chosen because higher chronic doses can cause the development of insulin resistance in animals [24]. In addition, we have previously shown that uridine at a dose of 30 mg/kg significantly mitigated damage to the myocardium of animals in the ischemia–reperfusion model [19].

Figure 1B–D shows the main somatic and biochemical characteristics of C57BL/6 mice in four experimental groups: (1) vehicle control (CTR); (2) uridine-treated control mice (CTR + U); (3) mice with high-fat diet–streptozotocin-induced diabetes mellitus (DM); and (4) DM mice treated with uridine (DM + U). One can see that DM mice did not differ significantly from control ones in terms of absolute heart weight or body weight. At the same time, we found that the heart weight/body weight ratio was significantly decreased in the DM group compared to the CTR one. The treatment of diabetic mice with uridine (DM + U group) led to a significant increase in this integral parameter to values comparable with those of control animals.

Figure 1E,F and Table 1 demonstrate the values of the selected blood biochemical indicators of experimental animals. According to the data obtained, diabetic animals showed a significant increase in the levels of plasma glucose, plasma triglycerides, and serum insulin, as well as a decrease in the rate of glucose utilization during the intraperitoneal glucose tolerance test (IPGTT; the total area under the curve (AUC) increased by 1.9 times compared to that in the CTR group). The administration of uridine to diabetic animals resulted in a significant reduction in plasma glucose and triglycerides, as well as an increase in the rate of glucose utilization according to the IPGTT (the AUC parameter decreased by 1.3 times compared to that in the DM group).

### 2.2. Effect of Uridine on the Ultrastructure of Heart Mitochondria in DM Mice

**Figure 2:**
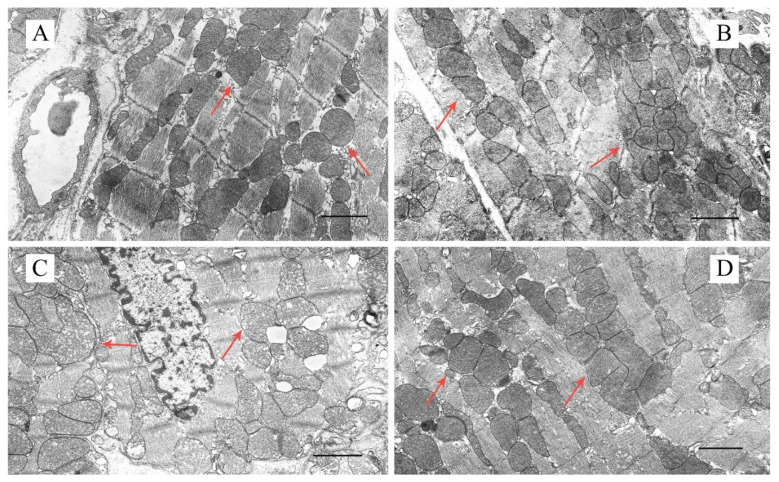
Representative transmission electron micrographs of mouse cardiomyocyte sections in the experimental groups: CTR (**A**), CTR + U (**B**), DM (**C**), and DM + U (**D**), showing the quality of the ultrastructure of cardiac intrafibrillar mitochondria. Images with ×5000 original magnification were taken from the basal inferior segment of the left ventricle of male C57BL/6NCrl mice. Red arrows indicate individual mitochondria. Scale bar: 1 μm.

**Figure 3:**
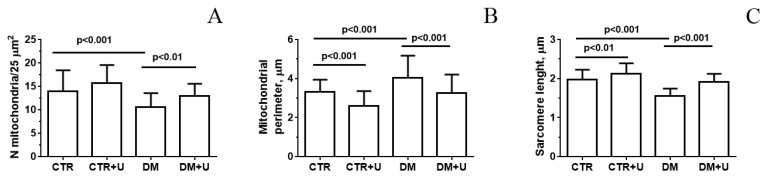
Ultrastructural analysis of the morphology of mouse cardiomyocytes in the experimental groups: the number of intrafibrillar mitochondria per field of view (25 μm^2^) (**A**), the perimeter of intrafibrillar mitochondria (**B**), and sarcomere length (**C**). The number of examined fields of view was 50–70 in each group. The values are given as means ± SD.

Figure 2, Figures S1 and S2 show representative electron micrographs (original magnification ×5000 or ×18,000) displaying the quality of the ultrastructure of mitochondria located between myofibrils (intrafibrillar mitochondria) and beneath the sarcolemma (subsarcolemmal mitochondria) in mouse ventricular cardiomyocytes in the experimental groups. As is known, a majority of the heart mitochondria are situated between myofibrils to meet the high energy demands of cardiac contractility. In the CTR and CTR + U groups, cardiac intrafibrillar mitochondria were characterized by an electron-dense matrix and contained a large number of densely packed and parallel-oriented cristae (Figure 2A,B and Figure S1A–D). Morphometric study revealed that the number of the mitochondria per field of view in these two groups did not differ (Figure 3A). At the same time, the size of the mitochondria (mitochondrial perimeter) was slightly reduced, and the length of the sarcomere was increased in the CTR + U group compared to the CTR group (Figure 3B,C). In the DM group, mitochondrial ultrastructure was substantially damaged (Figure 2C and Figure S1E,F). The organelles were either moderately or significantly swollen, with local areas of an enlightened matrix devoid of crista membranes. In some cases, the mitochondria exhibited vacuolization with the disruption of both the outer and inner membranes. The number of mitochondria per field of view in the DM group was reduced and their size was increased (Figure 3A,B). In addition, the sarcoplasm of diabetic cardiomyocytes contained enlarged cisterns of the sarcoplasmic reticulum and a reduced number of ribosomes and glycogen granules. The sarcomere length in the DM group was significantly less than in the CTR group (Figure 3C). Uridine treatment substantially restored the structural integrity of the mitochondria in diabetic cardiomyocytes (Figure 2D and Figure S1G,H). In the DM + U group, most intrafibrillar mitochondria had an electron-dense matrix with tightly arrayed cristae. The size of the mitochondria and their number were similar to those in the CTR group (Figure 3A,B). It should be noted that we observed similar changes in the subsarcolemmal subpopulation of mitochondria in the experimental groups (Figures S2 and S3). The sarcomere length in the DM + U group was increased compared to that in the DM group (Figure 3C).

### 2.3. Effect of Uridine on DM-Induced Changes in the mRNA Expression of Proteins Responsible for Mitochondrial Quality Control

**Figure 4:**
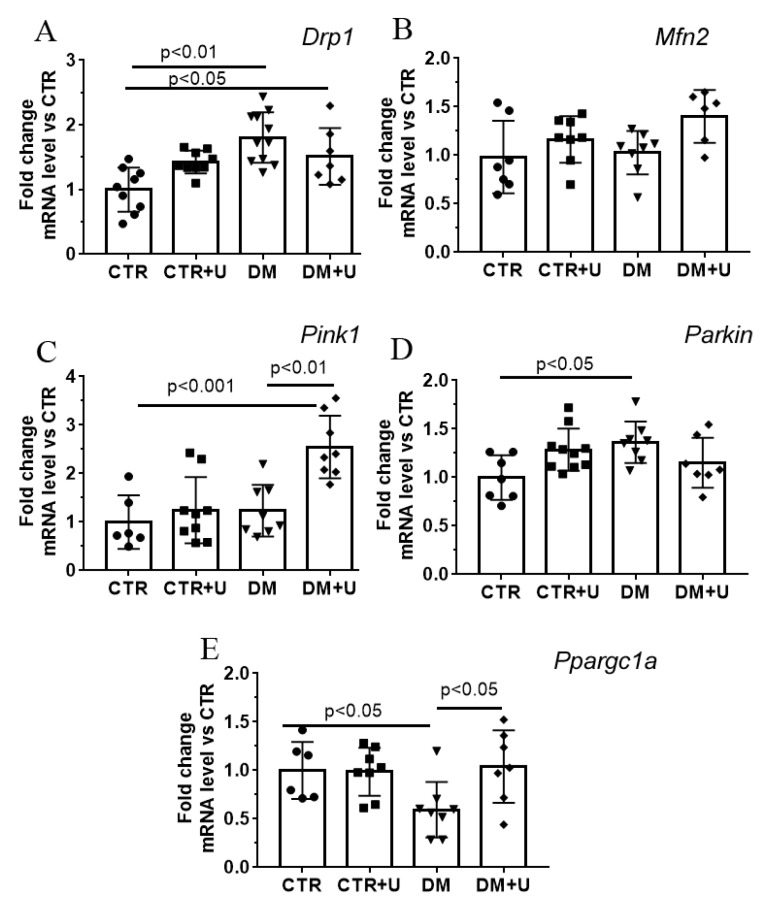
Effect of uridine treatment on mitochondrial homeostasis (the processes of mitochondrial fusion/fission, biogenesis, and mitophagy) in the diabetic heart. The relative mRNA levels of *Drp1* (**A**), *Mfn2* (**B**), *Pink1* (**C**), *Parkin* (**D**), and *Ppargc1a* (**E**) in the heart of mice in the experimental groups. The values are given as means ± SD (*n* = 6–10).

In the next part of the work, we determined changes in the level of expression of genes encoding proteins involved in mitochondrial fusion/fission, mitochondrial biogenesis, and mitophagy in the experimental groups (Figure 4). One can see that the development of DM in mice was accompanied by an increase in the mRNA level of *Drp1* (dynamin-related protein 1) and *Parkin*, suggesting the stimulation of the processes of mitochondrial fission and mitophagy, respectively. In parallel, the mRNA level of *Ppargc1a* (peroxisome proliferator activated receptor gamma coactivator 1 alpha) was decreased, which may indicate the suppression of mitochondrial biogenesis in the DM group.

Administration of uridine to diabetic mice normalized the expression of the *Ppargc1a* gene, but did not affect the expression of other genes studied. It should be noted that in the DM + U group, the mRNA level of *Pink1* (PTEN-induced putative kinase 1) increased, which may additionally indicate the activation of mitophagy. The mRNA level of the *Mfn2* (mitofusin 2) gene, which is involved in the mitochondrial fusion process, did not differ in any experimental group.

### 2.4. Effects of Uridine on DM-Induced Changes in the Functioning of Heart Mitochondria

Ultrastructural abnormalities and dysregulation of the quality-control system of mitochondria can promote dramatic changes in mitochondrial functions, the main one of which is the ability to produce ATP through oxidative phosphorylation. The DM group demonstrated a decrease in the rates of ADP- and 2,4-dinitrophenol (DNP)-stimulated respiration of the heart mitochondria (state 3 and 3U_DNP_, respectively) (Table 2). In parallel, we observed a decrease in the respiratory control ratio (RCR), indicating a reduction in the mitochondrial oxidative phosphorylation capacity in the DM group. Administration of uridine to diabetic mice resulted in a significant increase in ADP-driven state 3 respiration and the RCR parameter (while state 3U_DNP_ respiration only tended to restore). This suggests that uridine may contribute to the normalization of cardiomyocyte energy supply.

**Figure 5:**
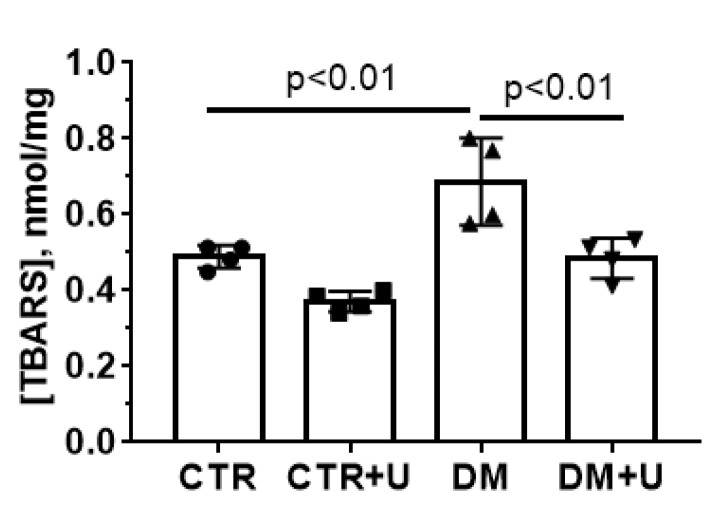
Uridine treatment suppresses the DM-induced lipid peroxidation in the heart mitochondria of experimental animals. Lipid peroxidation was assessed by the level of thiobarbituric acid-reactive substances (TBRAS) in the heart mitochondria of the experimental animals. All data are means ± SD (*n* = 4).

The oxidative damage to the heart mitochondria was next examined by the index of lipid peroxidation products (mainly malondialdehyde) using the thiobarbituric acid (TBA) assay (Figure 5). One can see that the level of TBA-reactive products in the mitochondria in the DM group increased significantly, while treatment of DM animals with uridine led to a decline in this indicator to control values.

**Figure 6:**
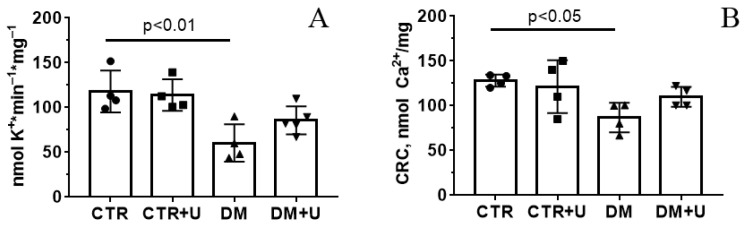
Assessment of the transport of potassium and calcium ions in the heart mitochondria of mice in the experimental groups. The rate of DNP-induced release of K^+^ from the heart mitochondria of experimental animals (**A**). Ca^2+^ retention capacity of the heart mitochondria of the experimental animals (**B**). The experimental conditions are described in the Section 4. All data are presented as means ± SD (*n* = 4–5).

Uridine is known to be a precursor of uridine 5′-diphosphate (UDP), an activator of the mitochondrial ATP-dependent potassium channel (mitoK_ATP_) [31]. In this regard, we evaluated the rate of DNP-induced release of K^+^ ions from mitochondria, which was mediated by the inversion of the mitoK_ATP_ [32]. As can be seen from Figure 6A, the development of DM was associated with the suppression of K^+^ release from the heart mitochondria. The administration of uridine to diabetic animals did not lead to a statistically significant increase in the rate of K^+^ release from the mitochondria (only a trend towards an increase in this parameter was observed).

As shown earlier, DM can be accompanied by a decrease in the resistance of heart mitochondria to the induction of the Ca^2+^-dependent PTP opening [8,33,34,35]. In the present work, we confirmed this observation. Figure 6B shows that the Ca^2+^ retention capacity (CRC) index was reduced in the DM group. Uridine treatment caused a 1.25-fold increase in this parameter, but this effect was statistically insignificant.

## 3. Discussion

Accumulating evidence suggests that the pyrimidine nucleoside uridine is essential for maintaining a number of cellular functions and energy metabolism [12]. The dynamics of changes in the concentration of uridine in plasma are part of the body’s systemic response to fasting and refeeding [20]. At the cellular level, the nucleoside is also involved in the regulation of carbohydrate, protein and lipid metabolism, the biosynthesis of nucleic acids, and also affects the functioning of mitochondria [12].

In this work, we demonstrate the protective effect of chronic administration of uridine against the development of mitochondrial dysfunction in the heart of C57BL/6 mice with DM induced by a high-fat diet combined with low-dose streptozotocin injections. According to the literature data, short-term administration of uridine to mice on a high-fat diet can significantly improve glucose tolerance [20]. Herein, we report that long-term treatment of diabetic mice with uridine (30 mg/kg/day for 21 days, i.p.) can also lead to an increase in the rate of glucose utilization from the blood of mice, as shown by the IPGTT (Figure 1). Our study demonstrates that the administration of uridine results in a decrease in the concentrations of glucose and triglycerides in the blood plasma of diabetic animals (Table 1). All this suggests that uridine facilitates the progression of diabetes. Similarly, some studies have shown that in diabetic patients, long-term administration of uridine, despite the absence of changes in the level of glycated hemoglobin, leads to a significant improvement in neurophysiological parameters [23].

The development of diabetic cardiomyopathy is one of the severe consequences of DM, which is defined as the occurrence of abnormal myocardial structure and function, with the exclusion of hypertension, coronary artery disease, and other preexisting cardiovascular conditions [4,5,6]. Diabetes-induced changes in cardiomyocytes include a wide range of structural and biochemical abnormalities that eventually lead to systolic and diastolic dysfunction and abnormal heart rate [34]. Interestingly, the development of DM is associated with a sharp decrease in the level of uridine in the heart [20], which may be one of the causes of violations of the structure and energy metabolism of the myocardium. Our previous work demonstrated that the administration of exogenous uridine to animals can prevent the development of myocardial injury and heart-rhythm disorders in acute ischemia and ischemia–reperfusion [16,17,18,19]. Here, we found that diabetes is accompanied by a significant decrease in the heart weight/body weight ratio, suggesting the development of diabetic cardiomyopathy, whereas uridine administration to animals with DM leads to the restoration of this indicator to control values.

Some studies revealed that the protective effect of uridine against ischemia-induced damage to the heart, as well as in some other cellular pathologies associated with oxidative stress, may be related to the normalization of the structure and function of mitochondria [19]. It was also demonstrated that pathological changes in mitochondria of the heart can appear earlier than clinical symptoms [36], indicating that mitochondrial dysfunction plays a crucial role in the pathogenesis of cardiovascular diseases. Based on these assumptions, we focused on the effect of uridine treatment on ultrastructural and coupled functional alterations in myocardial mitochondria of C57BL/6 mice with high-fat diet–streptozotocin-induced DM. Analysis of transmission electron micrographs showed that the structure of mitochondria of intrafibrillar and subsarcolemmal subpopulations in ventricular cardiomyocytes of diabetic mice was significantly damaged. In particular, the organelles displayed swelling, disruption of cristae membranes, and vacuolization. Ultrastructural analysis of the morphology of cardiac mitochondria of two subpopulations in the DM group revealed a reduction in their number and an increase in their size. We paid more attention to intrafibrillar mitochondria as the major mitochondrial subpopulation in cardiomyocytes [37] and since changes associated with myocardial insulin resistance seem to affect intrafibrillar mitochondria predominantly [38]. Some studies also showed that the intrafibrillar mitochondria are more sensitive to diabetes-related alterations [39], oxidative stress [40], and aging [41]. However, other authors found that subsarcolemmal mitochondria in type 2 diabetes were also impaired [42,43].

The administration of uridine (30 mg/kg/day) to diabetic mice for three weeks leads to an improvement in the morphology of the organelles in ventricular cardiomyocytes. In the DM + U group, the ultrastructure of the mitochondrial matrix and cristae, the size of mitochondria, and their number significantly differed from those in the DM group and were similar to those the CTR group. It is important to note that treatment with uridine leads not only to the preservation of mitochondrial ultrastructure but also to the normalization of other structures of cardiomyocytes. In particular, the DM + U group showed an increase in sarcomere length compared to the DM group.

Normalization of the ultrastructure and number of cardiomyocyte mitochondria in the case of uridine administration to diabetic animals may be associated with the restoration of systems responsible for mitochondrial homeostasis, namely, mitochondrial biogenesis, dynamics, and mitophagy. Indeed, we found that the DM group showed a decrease in the expression of the *Ppargc1a* gene responsible for the synthesis of the PGC1α protein, the main regulator of mitochondrial biogenesis. Administration of uridine to diabetic mice resulted in a significant increase in the level of *Ppargc1a* mRNA. This may indicate the normalization of mitochondrial biogenesis in cardiomyocytes of the DM + U group. We did not observe a significant change in *Drp1* and *Parkin* mRNA levels after uridine treatment, which are responsible for the synthesis of Drp1 and Parkin proteins involved in the processes of mitochondrial fission and mitophagy. Nevertheless, the DM + U group showed an increase in the level of *Pink1* mRNA (the PINK1 protein is also involved in mitophagy). This suggests that uridine stimulates mitophagy in diabetic animals. The increased exclusion of defective mitochondria along with stimulation of mitochondrial biogenesis by uridine may lead to the occurrence of a sufficient population of healthy mitochondria in diabetic cardiomyocytes. It should be noted that although the changes observed in the mRNA levels of the proteins studied are indirect evidence, they are fully consistent with the literature data that diabetes mellitus is accompanied by an increase in mitochondrial fission and a decrease in mitochondrial biogenesis and mitophagy [8].

Indeed, as shown in Table 2 and in Figure 5, diabetic animals demonstrated a decrease in the rate of respiration in state 3, the respiratory control ratio, and an increase in the content of TBARS in the heart mitochondria, while uridine treatment prevented bioenergetic defects and oxidative damage to the organelles. A similar effect of uridine was observed earlier in models of acute myocardial ischemia and ischemia–reperfusion [19].

Mitochondrial dysfunction in DM is mediated not only by a violation of oxidative phosphorylation and excessive production of ROS but also by the dysregulation of ion homeostasis [8]. As shown in this work, the DM group showed a reduction in the rate of transport of K^+^ in the mitochondria of the heart. It is believed that the transport of K^+^ through the inner mitochondrial membrane is mediated by the mitoK_ATP_ channel, which can be activated by UDP and whose metabolic precursor is uridine [31,44,45]. We could not detect a significant increase in the rate of transport of K^+^ in the DM + U group compared to the DM group (only an upward trend was observed). One can suggest that this concentration of uridine is not sufficient to restore this parameter in diabetic mice.

The development of DM is accompanied by a decrease in the resistance of mitochondria of the cardiac and skeletal muscles to the PTP opening [33,34,35,46,47,48]. It is known that the PTP is a multiprotein channel formed in the inner and outer mitochondrial membranes in the development of oxidative stress, increased Ca^2+^ concentration, and a number of other stimuli. The main structural components of the PTP are cyclophilin D, adenylate translocator, and ATP synthase [49,50]. The opening of this pore in mitochondria leads to the collapse of the mitochondrial membrane potential, the dysregulation of calcium ion homeostasis, and the destruction of the organelles [50]. In previous studies, we have demonstrated that the treatment of mice with DM with the PTP blocker alisporivir or the administration of the drug in primary mouse endothelial cells under conditions of hyperglycemia leads to an improvement in the biochemical parameters and functional characteristics of mitochondria [34,46,47]. The administration of uridine to diabetic animals does not affect the CRC parameter, reflecting the resistance of mitochondria to the induction of the Ca^2+^-dependent PTP opening. Thus, under our experimental conditions, uridine was unable to inhibit the formation of the PTP in diabetic mitochondria. Taken together, our findings suggest that, along with its glucose-lowering effect, uridine has a protective action against diabetes-mediated functional and structural damage to cardiac mitochondria and disruption of mitochondrial quality-control systems in the diabetic heart.

## 4. Materials and Methods

### 4.1. Experimental Animals, Induction and Validation of Diabetes

Male mice of the C57BL/6NCrl line weighing 22–24 g were used. The animals were purchased from the Animal Breeding Facility, Branch of the Shemyakin and Ovchinnikov Institute of Bioorganic Chemistry, Russian Academy of Sciences, (IBCh RAS Unique Research Device “Bio-model”, Pushchino, Russia). The mice were randomly divided into four experimental groups: (1) vehicle-treated control (CTR) (*n* = 10); (2) control + uridine (CTR + U) (*n* = 10); (3) DM (*n* = 10); and (4) mice with DM treated with uridine (DM + U) (*n* = 10). The induction of diabetes in experimental mice was carried out using a combination of a high-fat diet with multiple injections of low doses of streptozotocin according to the protocol described in our previous work [51]. The scheme for induction of DM and diabetes tests is presented in Figure 1. It should be noted that the mouse model of DM developed using a high-fat diet combined with low-dose streptozotocin injections recreates the major pathophysiological features of diabetes in patients [52,53]. The mice in the CTR + U and DM + U groups were treated with uridine at a dose of 30 mg/kg per day via i.p. injection for 21 days. Uridine (Sigma-Aldrich, St. Louis, MO, USA) was dissolved in sterile saline immediately prior to injections. The control mice were treated with vehicle alone. The successful induction of DM was confirmed by the intraperitoneal glucose tolerance test (IPGTT). For the test, a stock solution of glucose (2 g/kg) in 0.1 mL of distilled water was administered intraperitoneally. The blood glucose level was recorded using a One Touch Select Plus glucometer (LifeScan, Zug, Switzerland). The triglyceride level in the blood of mice was estimated using a Multicare-In biochemistry analyzer (Biochemical Systems International Srl, Arezzo, Italy). Serum insulin level was measured spectrophotometrically using a mouse insulin solid-phase sandwich ELISA kit (Thermo Fisher Scientific, Waltham, MA, USA) according to the manufacturer’s recommendations.

### 4.2. Transmission Electron Microscopy

Samples of the heart tissue (the basal inferior segment of the left ventricle) for the transmission electron microscopy (TEM) imaging were taken from decapitated animals and fixed for 2 h in a 2.5% glutaraldehyde solution in 0.1 M PBS (pH = 7.4) [54]. The preparations were examined and photographed using a JEM-100B electron microscope (JEOL, Tokyo, Japan) at original magnifications of ×5000 and ×18,000. The results of the TEM analysis were presented as representative images from two biological replicates. The number of examined fields of view was 50–70 in each group. The data obtained were analyzed using the Image Tool 3.0 software.

### 4.3. RNA Extraction, Reverse Transcription, and Quantitative Real-Time PCR

Total RNA was isolated from 100 mg of deep-frozen tissue samples from the quadriceps using an ExtractRNA kit (#BC032, Eurogen, Moscow, Russia) in accordance with the protocols of the manufacturer. Real-time PCR was performed on a QuantStudio 1 (Thermo Fisher Scientific, USA) using the qPCRmix-HS SYBR reaction mixture (Eurogen, Moscow, Russia). The selection and analysis of gene-specific primers were performed using Primer-BLAST (the oligonucleotide sequences are presented in Table 3). The relative expression level of each gene was normalized to the level of *Rplp2* mRNA, and a comparative C_T_ method was used to quantify the results [55].

### 4.4. Isolation of Heart Mitochondria and Assessment of Mitochondrial Functions

Mitochondria were isolated from fresh mouse heart tissue by differential centrifugation [56]. Final suspensions contained 15–30 mg of mitochondrial protein/mL, as determined using a Bradford Assay Kit (ab102535, Abcam, Cambridge, UK). The rate of O_2_ consumption by isolated mitochondria was estimated by high-resolution respirometry with the Oroboros Oxygraph-2k (Oroboros Instruments, Innsbruck, Austria) [57]. The reaction buffer contained 120 mM KCl, 5 mM NaH_2_PO_4_, 2.5 mM potassium malate, 2.5 mM potassium glutamate, and 10 mM HEPES/KOH (pH 7.4). Ca^2+^ transport in mitochondria was estimated with an Arsenazo III indicator at 675–685 nm using a Tecan Spark 10M plate reader (Tecan Group Ltd., Männedorf, Switzerland) [56]. The reaction buffer contained 210 mM mannitol, 70 mM sucrose, 1 mM KH_2_PO_4_, 2.5 mM malate, 2.5 mM glutamate, 10 μM EGTA, 50 μM Arsenazo III, and 10 mM HEPES-KOH (pH 7.4.). The total amount of the added Ca^2+^ ions that induced their spontaneous release from mitochondria due to the induction of the PTP opening was interpreted as the calcium retention capacity (CRC) index of the organelles. Transport of K^+^ in mitochondria was determined by the rate of DNP-induced release of K^+^ from the organelles [32]. The DNP-induced K^+^ efflux was measured with an ion-selective electrode (Nico-Analyte LLC, Moscow, Russia). The incubation medium contained 180 mM sucrose, 70 mM mannitol, 5 mM NaH_2_PO_4_, 1 μg/mL oligomycin, and 10 mM Tris/HCl, pH 7.4. Intensity of lipid peroxidation in mitochondrial membranes was assessed spectrophotometrically by quantification of thiobarbituric acid-reactive substances represented by malondialdehyde and some other minor aldehyde species, as described in [34].

### 4.5. Statistical Analysis

The data were analyzed using the GraphPad Prism 7.0 software (GraphPad Software Inc., San Diego, CA, USA) and are presented as means ± SD of 4–10 biological replicates (excluding electron microscopy data). We used the Shapiro–Wilk normality test to verify that the data had Gaussian distribution. If the data fit the normal distribution, the statistical significance of the differences between the experimental groups was analyzed with one-way analysis of variance (ANOVA), followed by the Tukey multiple comparison post hoc test. For not normally distributed data, one-way ANOVA using the Kruskal-Wallis test were used. *p*-values < 0.05 were considered statistically significant.

## 5. Conclusions

In the present work, we investigated the effect of long-term administration of uridine (30 mg/kg/day for 3 weeks) on the course of DM and its concomitant mitochondrial dysfunction in heart tissue in the high-fat diet–low-dose streptozotocin-induced diabetic mouse model. The following results were obtained: (1) uridine treatment increased the rate of glucose utilization during the IPGTT in the DM group; (2) uridine normalized the ultrastructure and the number of mitochondria in ventricular cardiomyocytes of diabetic mice; (3) uridine administration to diabetic mice restored the mRNA level of *Ppargc1a* and enhanced *Pink1* gene expression, which may indicate an increase in the intensity of mitochondrial biogenesis and mitophagy, and as a consequence mitochondrial turnover; and (4) uridine restored the mitochondrial oxidative phosphorylation capacity and prevented oxidative damage to the organelles, but did not affect the systems responsible for mitochondrial ion transport in the hearts of mice with DM. As mentioned above, diabetic mice demonstrated a significant decrease in uridine levels in blood plasma, bile, heart, and brown adipose tissue [20]; therefore, the administration of uridine can maintain its levels in the tissues and normalize the energy metabolism of cardiomyocytes. It is important to note that in this work, we administered low doses of uridine, which did not lead to adverse effects on DM parameters or mitochondrial function. The results obtained allow us to characterize the action of uridine as a drug capable of reducing the systemic consequences of diabetes and preventing the disruption of mitochondrial homeostasis coupled with bioenergetic defects and oxidative stress in the hearts of mice with experimental diabetes. Further studies are needed to determine the effect of uridine on other diabetic organs and tissue (for example, the liver), in which its level does not decrease with the progression of diabetes mellitus.

## Figures and Tables

**Table 1:** Biochemical characteristics of the blood of mice in the experimental groups.

	CTR	CTR + U	DM	DM + U
BG (fed state), mM	10.1 ± 0.2	9.2 ± 0.7	14.8 ± 0.6 *	11.3 ± 1.5 #
Triglycerides, mM	1.56 ± 0.05	1.63 ± 0.15	2.18 ± 0.24 *	1.34 ± 0.25 #
Insulin, μIU/mL	14.0 ± 0.2	14.7 ± 0.3	15.9 ± 0.3 *	15.0 ± 0.4

Values are given as means ± SEM (*n* = 10). BG, blood glucose. * *p* < 0.05 compared to the control group (CTR). # *p* < 0.05 compared to the DM group.

**Table 2:** Parameters of respiration and oxidative phosphorylation of mouse heart mitochondria in the experimental groups.

Group	V Respiration, nmol O_2_ * min^−1^ * mg^−1^ Protein				RCR
	State 2	State 3	State 4	State 3U_DNP_	
CTR	18.3 ± 1.7	45.9 ± 1.9	18.6 ± 0.6	43.9 ± 3.8	2.5 ± 0.1
CTR + U	15.5 ± 0.7	41.8 ± 1.3	15.1 ± 1.3	41.7 ± 1.9	2.8 ± 0.2
DM	13.5 ± 0.9	29.6 ± 0.4 *	15.4 ± 0.3	28.9 ± 1.5 *	1.9 ± 0.1 *
DM + U	16.8 ± 1.1	36.3 ± 1.1 *#	14.0 ± 0.4 *	35.0 ± 1.1 *	2.6 ± 0.1 #

Mitochondria respiration was fueled by 2.5 mM glutamate and 2.5 mM malate. State 3 respiration was initiated by 100 µM ADP. The results are presented as means ± SEM (*n* = 6). * *p* < 0.05 compared to the control group (CTR). # *p* < 0.05 compared to the DM group.

**Table 3:** List of gene-specific primers for RT-PCR analysis.

Gene	Forward (5′→3′)	Reverse (5′→3′)
*Pink1*	TTGCCCCACACCCTAACATC	GCAGGGTACAGGGGTAGTTCT
*Parkin*	AGCCAGAGGTCCAGCAGTTA	GAGGGTTGCTTGTTTGCAGG
*D* *rp1*	TTACAGCACACAGGAATTGT	TTGTCACGGGCAACCTTTTA
*Mfn2*	CACGCTGATGCAGACGGAGAA	ATCCCAGCGGTTGTTCAGG
*Ppargc1a*	CTGCCATTGTTAAGACCGAG	GTGTGAGGAGGGTCATCGTT
*Rplp2*	CGGCTCAACAAGGTCATCAGTGA	AGCAGAAACAGCCACAGCCCCAC

## Data Availability

The data presented in this study are available upon request from the corresponding author.

## Supplementary Materials

The supporting information can be downloaded at: https://www.mdpi.com/article/10.3390/ijms231810633/s1.
