# Supplementary material for: Effect of Chronic Treatment with Uridine on Cardiac Mitochondrial Dysfunction in the C57BL/6 Mouse Model of High-Fat Diet–Streptozotocin-Induced Diabetes
Source: Int J Mol Sci. 2022 Sep 13;23(18):10633. doi: 10.3390/ijms231810633 (PMC9502122; doi:10.3390/ijms231810633)
Supplement: Supplementary file 1 [file ijms-23-10633-s001.zip › ijms-1890629-supplementary.pdf]

Supplementary Materials to the ijms-1890629 manuscript

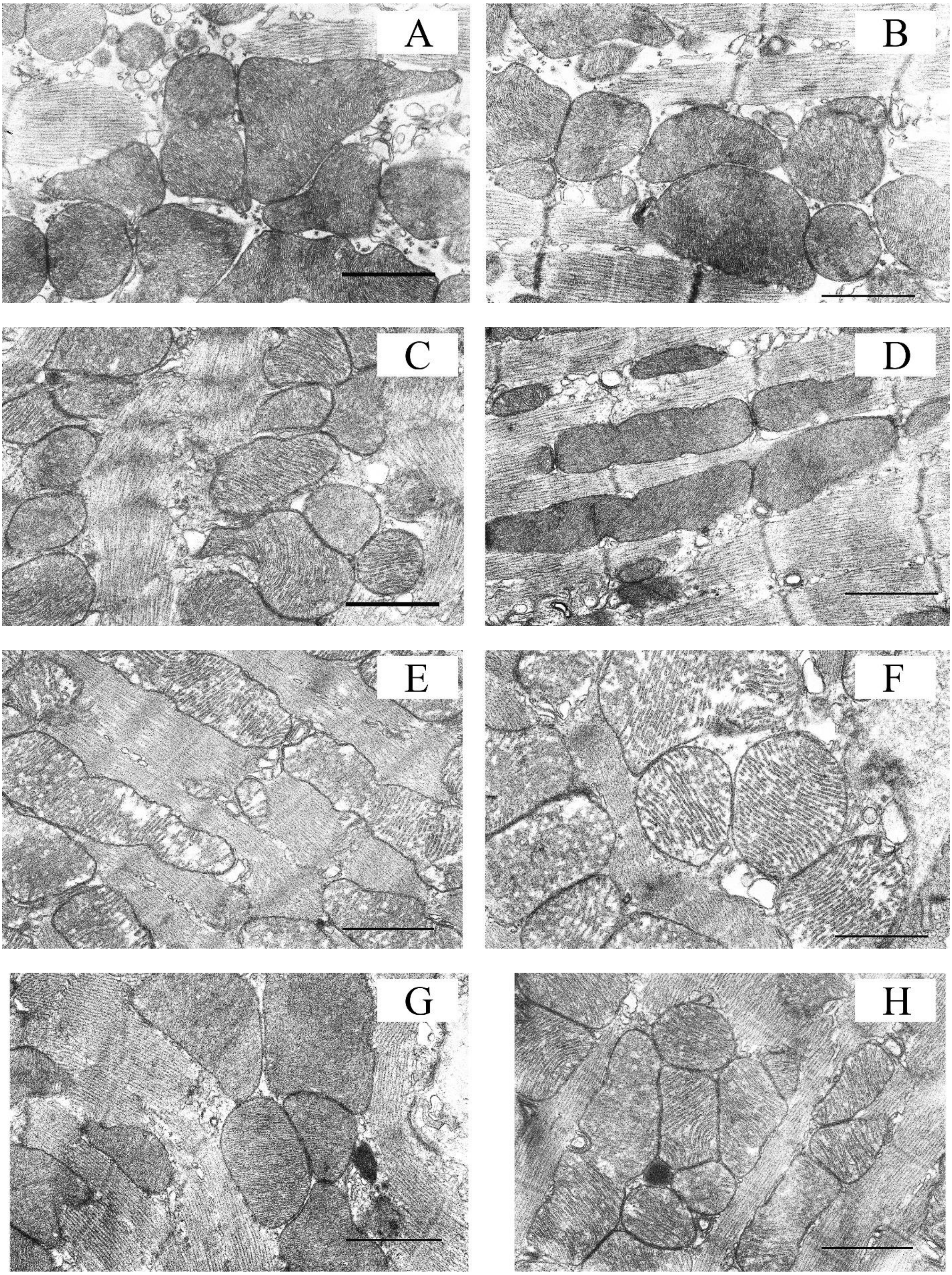

Figure S1: Representative micrographs with a higher original magnification (x18,000), displaying individual intrafibrillar mitochondria in mouse ventricular cardiomyocytes in the experimental groups: CTR (**A**-**B**), CTR+U (**C**-**D**), DM (**E**-**F**), and DM+U (G-**H**). The outer and inner mitochondrial membranes, matrix and cristae are clearly visible. Scale bar: 1 μm.

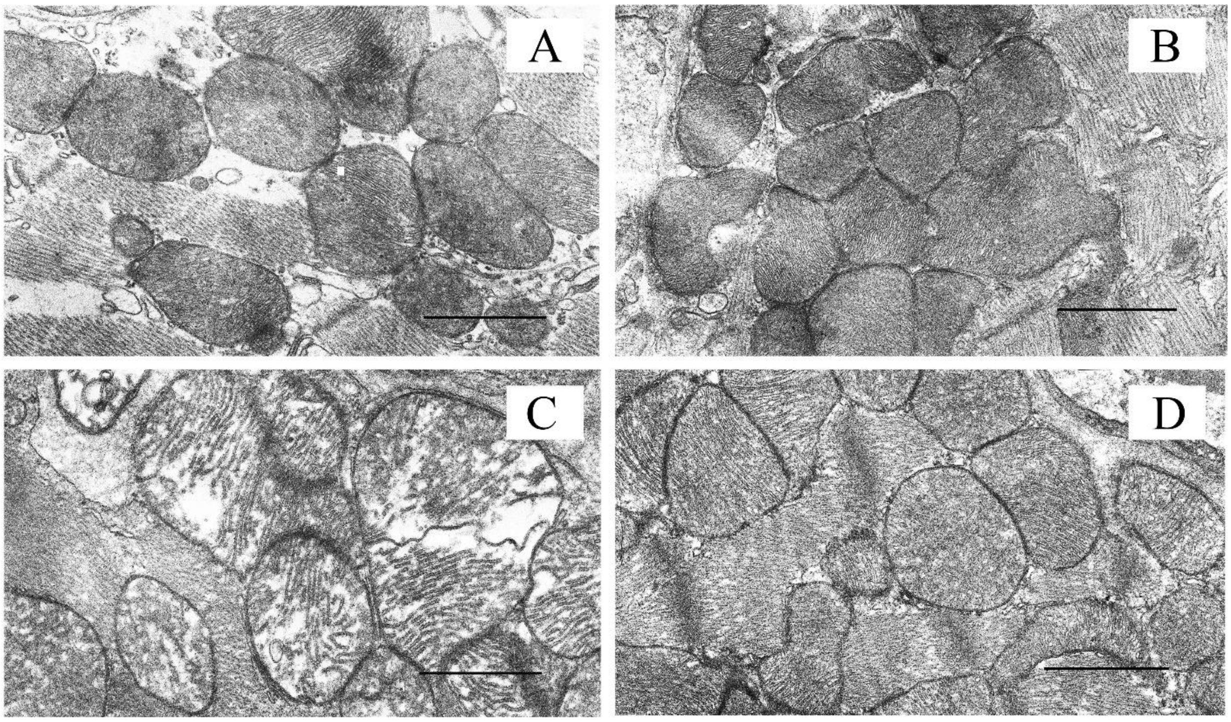

Figure S2: Representative TEM images with a higher original magnification (× 18,000), displaying subsarcolemmal mitochondria in mouse ventricular cardiomyocytes in the experimental groups: CTR (**A**), CTR+U (B), DM (**C**), and DM+U (**D**).

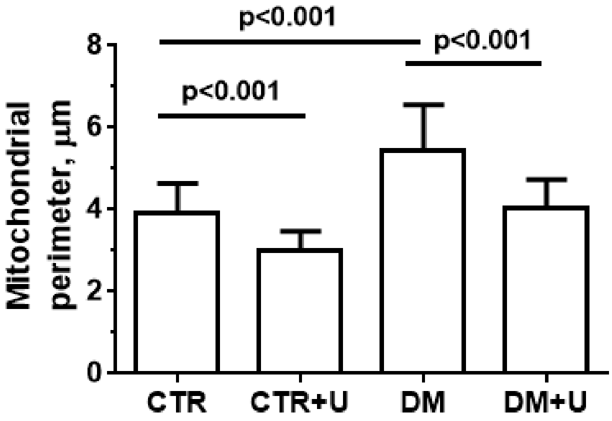

Figure S3: The perimeter of the subsarcolemmal mitochondria in mouse cardiomyocytes in the experimental groups. The number of examined fields of view was 30–50 in each group. The values are given as means ± SD.
